# Efficacy and Safety of Oral Orforglipron for Weight Reduction in Adults With Overweight or Obesity: A Systematic Review

**DOI:** 10.7759/cureus.117774

**Published:** 2026-10-11

**Authors:** Gabriel Jose Paredes Yacaman, Ariadna Montzerrat Jiménez López, Paola Robles Cordova, Sebastián E Abad Cuenca, Stephanie Mora López, Alfonso Iker Cobos Lladó, Diego Oliverio Bautista Quintero, Edgar David Castillo Bárcena, Raúl Antonio Zamarripa Mottú, Victor Zúñiga Flores, Jose R Flores Valdés, Mauricio Montelongo Quevedo

**Affiliations:** 1 Medicine, Universidad Autónoma de Guadalajara, Guadalajara, MEX; 2 General Practice, Universidad Nacional Autónoma de México (UNAM), Mexico City, MEX; 3 Division of Geriatrics and Palliative Medicine, Department of Internal Medicine, The University of Texas Health Science Center at Houston, Houston, USA; 4 Internal Medicine, Consultorios Médicos de Especialidades "San Miguel", Azogues, ECU; 5 Internal Medicine, Hospital General de Zona con Medicina Familiar No. 29 'Aragon', Mexico City, MEX; 6 Internal Medicine, Hospital General de Occidente, Zapopan, MEX; 7 Internal Medicine, Hospital Regional de Alta Especialidad de la Peninsula de Yucatan, Merida, MEX; 8 Internal Medicine, Hospital Juárez de México, Mexico City, MEX; 9 Gastroenterology, Hospital de Especialidades, Centro Médico Nacional Siglo XXI, Mexico City, MEX; 10 Gastroenterology, Unidad de Atención Médica Ambulatoria (UMAA) No. 90, Instituto Mexicano del Seguro Social, Torreón, MEX; 11 Gastroenterology, Hospital Angeles Torreón, Torreón, MEX; 12 Diagnostic Laboratory Hematology, Laboratorio Flores, Nuevo Laredo, MEX; 13 Thoracic Head and Neck Medical Oncology, MD Anderson Cancer Center, Houston, USA; 14 General Medicine, Universidad Autónoma de Guadalajara, Guadalajara, MEX

**Keywords:** diabetes, glp1 analogues, obesity, obesity and diabetes, obesity and overweight, orforglipron, overweight, weight reduction

## Abstract

Obesity is a chronic disease associated with increased cardiometabolic risk, reduced quality of life, and higher mortality. Although injectable glucagon-like peptide-1 receptor agonists (GLP-1RAs) have substantially improved weight management, the requirement for injections remains a limitation for many patients. Orforglipron is a novel nonpeptide oral GLP-1RA currently under investigation for the treatment of overweight, obesity, and type 2 diabetes mellitus (T2DM). This systematic review evaluated the efficacy and safety of oral orforglipron in adults with overweight or obesity, with or without T2DM. A structured literature search was conducted using PubMed and ScienceDirect from database inception through December 2025. Randomized controlled trials evaluating oral orforglipron and reporting weight-related, glycemic, or metabolic outcomes were included. Six randomized controlled trials involving 6,022 participants were included in the qualitative synthesis, with follow-up durations ranging from 12 to 72 weeks. Across studies, treatment with orforglipron was associated with clinically meaningful reductions in body weight ranging from approximately −4.5% to −14.7%, with greater reductions generally observed in longer-duration studies and at higher doses. Improvements in glycated hemoglobin (HbA1c), fasting glucose, waist circumference, systolic blood pressure, triglycerides, and non-high-density lipoprotein cholesterol were also reported across several studies. Gastrointestinal adverse events, particularly nausea, vomiting, diarrhea, and constipation, were the most commonly reported safety outcomes and occurred more frequently during dose escalation and in higher-dose groups. Overall, current evidence suggests that oral orforglipron may represent a promising therapeutic option for weight reduction and glycemic improvement in adults with overweight or obesity; however, additional long-term randomized controlled trials are needed to further clarify optimal dosing strategies, long-term safety, and comparative clinical effectiveness.

## Introduction and background

Obesity is a chronic condition associated with increased cardiometabolic risk, reduced quality of life, and increased morbidity and mortality worldwide [1]. Despite important advances in obesity management, the development of effective, accessible, and well-tolerated pharmacological therapies remains an important clinical priority [1]. Glucagon-like peptide-1 (GLP-1) receptor agonists (GLP-1RAs) and other incretin-based therapies have transformed the treatment landscape by producing clinically meaningful reductions in body weight and improvements in metabolic outcomes [2].

However, many highly effective GLP-1-based therapies require subcutaneous administration, which may represent a barrier for some patients and affect long-term treatment acceptance. Although oral GLP-1RA formulations have been developed, there remains a need for effective oral alternatives capable of providing substantial weight reduction with acceptable tolerability profiles [2]. Orforglipron is a non-peptide, small-molecule oral GLP-1 receptor agonist designed to activate GLP-1 signaling pathways, enhancing glucose-dependent insulin secretion, delaying gastric emptying, and increasing satiety, thereby contributing to improvements in glycemic control and body weight reduction [3].

Given the increasing burden of obesity and the rapid expansion of pharmacologic treatment options, a rigorous synthesis of emerging evidence is essential to inform clinical decision-making and guide therapeutic selection. A previous systematic review and meta-analysis evaluated early clinical evidence regarding orforglipron; however, available data were limited by the number of included trials, sample size, and follow-up duration [3]. The publication of additional randomized controlled trials with larger study populations and longer follow-up periods supports the need for an updated synthesis of the efficacy and safety of orforglipron [3].

Therefore, we conducted a systematic review of randomized controlled trials to evaluate the efficacy and safety of oral orforglipron in adults with overweight or obesity, with or without type 2 diabetes mellitus (T2DM). The primary research question was: In adults with overweight or obesity, does orforglipron reduce body weight (percent change and clinically relevant weight-loss thresholds), and what is its associated safety and tolerability profile? To address this question, we systematically searched electronic databases for randomized controlled trials, extracted efficacy and safety outcomes, and assessed risk of bias.

## Review

Methods

This study adhered to the Preferred Reporting Items for Systematic Reviews and Meta-Analyses (PRISMA) 2020 guidelines and followed principles of evidence-based medicine to ensure a comprehensive and systematic approach to the review [4,5]. The protocol was registered in the International Prospective Register of Systematic Reviews (PROSPERO) database (CRD420261384144). 

Objective

The objective of this systematic review is to evaluate the efficacy and safety of oral orforglipron for weight reduction in adults with overweight or obesity, with or without type 2 diabetes.

Search Methods

To ensure the selection of relevant studies, predefined eligibility criteria were applied before study screening. Articles that did not meet the established inclusion criteria, did not evaluate oral orforglipron, or lacked accessible full-text data were excluded.

A comprehensive literature search was conducted using electronic databases, including PubMed and ScienceDirect, to identify studies evaluating the efficacy and safety of oral orforglipron in adults with overweight or obesity, with or without T2DM. The search strategy incorporated relevant Medical Subject Headings (MeSH) terms and free-text keywords associated with oral orforglipron, obesity, weight reduction, and type 2 diabetes. Databases were searched from inception through December 2025, corresponding to the date of the final search, and only studies published in English were considered eligible. In addition, the reference lists of included studies and relevant review articles were manually screened to identify potentially eligible studies not captured through the electronic database search. The study selection process followed a structured approach consistent with PRISMA 2020 recommendations.

Selection Criteria

Types of participants: For this systematic review, we established selection criteria that included adults aged ≥18 years with overweight or obesity, including populations with or without T2DM, who were enrolled in clinical studies evaluating oral orforglipron. Studies including participants of both sexes and any racial or ethnic background were considered eligible. We excluded studies involving pediatric populations, pregnant individuals, healthy volunteers, and populations with acute illness or advanced comorbidities inconsistent with obesity pharmacotherapy trials.

Types of intervention: This systematic review focused on daily oral orforglipron administered using clinically investigated dose regimens in adults with overweight or obesity. Eligible studies were required to include at least one oral orforglipron treatment arm and report relevant weight-related or glycemic outcomes. Non-oral formulations or studies not specifically evaluating oral orforglipron were excluded.

Types of studies: A systematic search was conducted for relevant research articles published in English from database inception through December 2025. The selection focused on randomized controlled trials evaluating the efficacy and safety of oral orforglipron. To maintain methodological rigor, observational studies, case reports, narrative reviews, conference abstracts, editorials, preclinical studies, and early phase 1 studies conducted exclusively in healthy volunteers were excluded.

Type of outcomes: The primary outcome of this review was percent change in body weight. Secondary outcomes included the proportion of participants achieving clinically significant weight-loss thresholds (5%, 10%, and 15%), changes in body mass index and waist circumference, glycemic outcomes when applicable, and adverse events, including gastrointestinal and severe adverse events.

Selection Process and Data Collection

All records identified through the database search were imported into Rayyan (Rayyan Systems, Inc., Cambridge, MA) for screening and management of study selection [6]. Two reviewers (EC and SA) independently screened titles and abstracts according to the predefined eligibility criteria, followed by full-text assessment of potentially eligible studies. A third reviewer verified the relevance of the studies and assisted in resolving uncertainties during the screening process. Two additional reviewers (SL and RZ) independently evaluated full-text articles using the same inclusion and exclusion criteria. Disagreements at any stage of the selection process were resolved through discussion and consensus, with adjudication by a third reviewer when necessary.

Two reviewers (SA and AJ) independently extracted data from each included study using a standardized data extraction form developed a priori to ensure consistency and minimize bias throughout the review process. Extracted variables included study characteristics (author, year, country, and study design), participant demographics, baseline clinical characteristics, intervention dose, comparator groups, treatment duration, and reported efficacy and safety outcomes. Particular attention was given to outcomes related to percent change in body weight, categorical weight-loss responses, anthropometric measures, glycemic parameters when applicable, and adverse events. Any discrepancies during data extraction were resolved through discussion and consensus, with adjudication by a third reviewer (PR) when necessary. No automation tools or machine learning classifiers were used during screening or data extraction, and study authors were not contacted for additional information because all relevant data were available within the published reports. Additionally, no translations were required, as only studies published in English were considered eligible. When multiple publications referred to the same clinical trial, the available data were reviewed collectively and treated as a single study to avoid duplication.

Data Synthesis

The extracted findings were synthesized narratively and organized according to the principal outcome domains evaluated in the review, including body weight reduction, categorical weight-loss responses, glycemic outcomes, and safety. Quantitative findings reported by the individual trials were summarized descriptively at the study-specific follow-up time points. Given the clinical and methodological differences across the included trials, no formal quantitative meta-analysis or pooled statistical analysis was performed.

Risk of Bias Assessment

The methodological quality of the included studies was evaluated according to the recommendations outlined in the Cochrane Handbook for Systematic Reviews of Interventions [7]. Two reviewers (PR and AJ) independently assessed the risk of bias of each included randomized controlled trial using the Cochrane Risk of Bias 2 (RoB 2) tool [8]. The RoB 2 assessment was conducted for the primary outcome of percent change in body weight and evaluated the effect of assignment to intervention. Domain-level judgments were determined using the corresponding RoB 2 signaling questions. The assessment included the following domains: bias arising from the randomization process, bias due to deviations from intended interventions, bias resulting from missing outcome data, bias in the measurement of outcomes, and bias in the selection of the reported results.

Each domain was classified as low risk, some concerns, or high risk of bias, and an overall judgment was assigned to each study according to Cochrane recommendations. Any disagreements between reviewers were resolved through discussion and consensus, with consultation from a third reviewer (GP) when necessary. No modifications were made to the original RoB 2 tool, and no automation tools were used during the assessment process. Study authors were not contacted for additional methodological information.

Results

Study Selection

A structured literature review was conducted to evaluate the efficacy and safety of oral orforglipron in adults with overweight or obesity. The search was performed from database inception through December 2025 using electronic databases including PubMed and ScienceDirect. A targeted search strategy was applied using relevant keywords related to “orforglipron,” “obesity,” “weight loss,” and “adults.”

The initial search identified 162 records. After the removal of duplicate and ineligible records prior to screening, 45 articles remained for title and abstract screening, resulting in the exclusion of 12 records. Subsequently, 33 full-text articles were assessed for eligibility, of which 27 were excluded due to ineligible study design, population mismatch, or insufficient or irrelevant outcome data. Ultimately, six randomized controlled trials fulfilled all predefined eligibility criteria and were included in the final qualitative synthesis.

The study selection process is illustrated in Figure 1 using the PRISMA 2020 flow diagram, which enhances the transparency and reproducibility of the screening and selection process [9].

**Figure 1:**
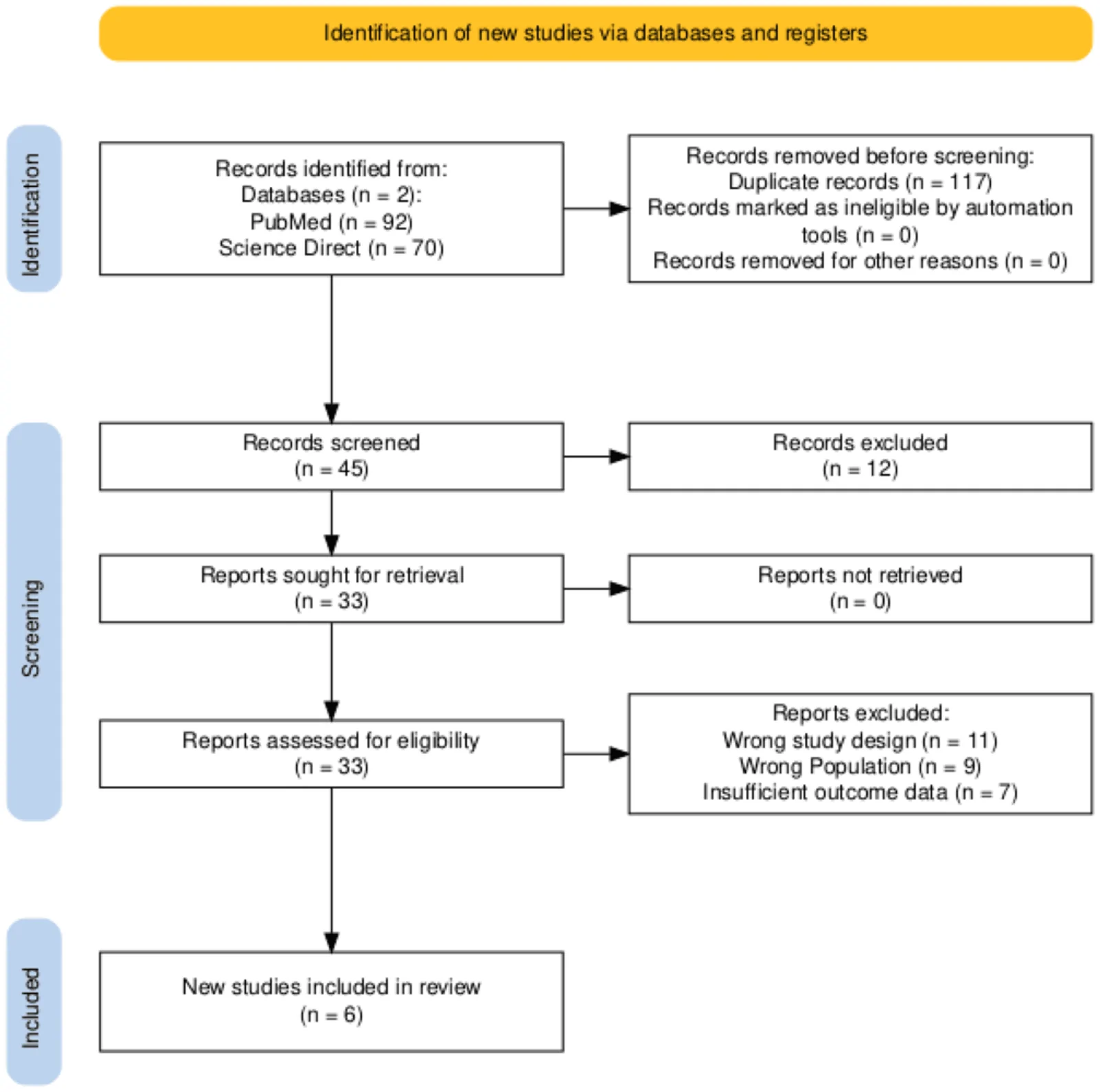
PRISMA 2020 flow diagram of study selection. This diagram details the step-by-step process of article screening.

Risk of Bias in Studies

The risk of bias assessment, performed using the Cochrane RoB 2 tool, demonstrated generally favorable methodological quality across the six included randomized controlled trials (Figure 2, 3) [8,10]. Figure 2 presents the individual risk-of-bias judgments for each included study, whereas Figure 3 summarizes the overall distribution of risk-of-bias judgments across the six included randomized controlled trials. Four studies (67%) were judged to have an overall low risk of bias, while two studies (33%) were classified as having some concerns. No included study was considered to have a high overall risk of bias [11-16].

**Figure 2:**
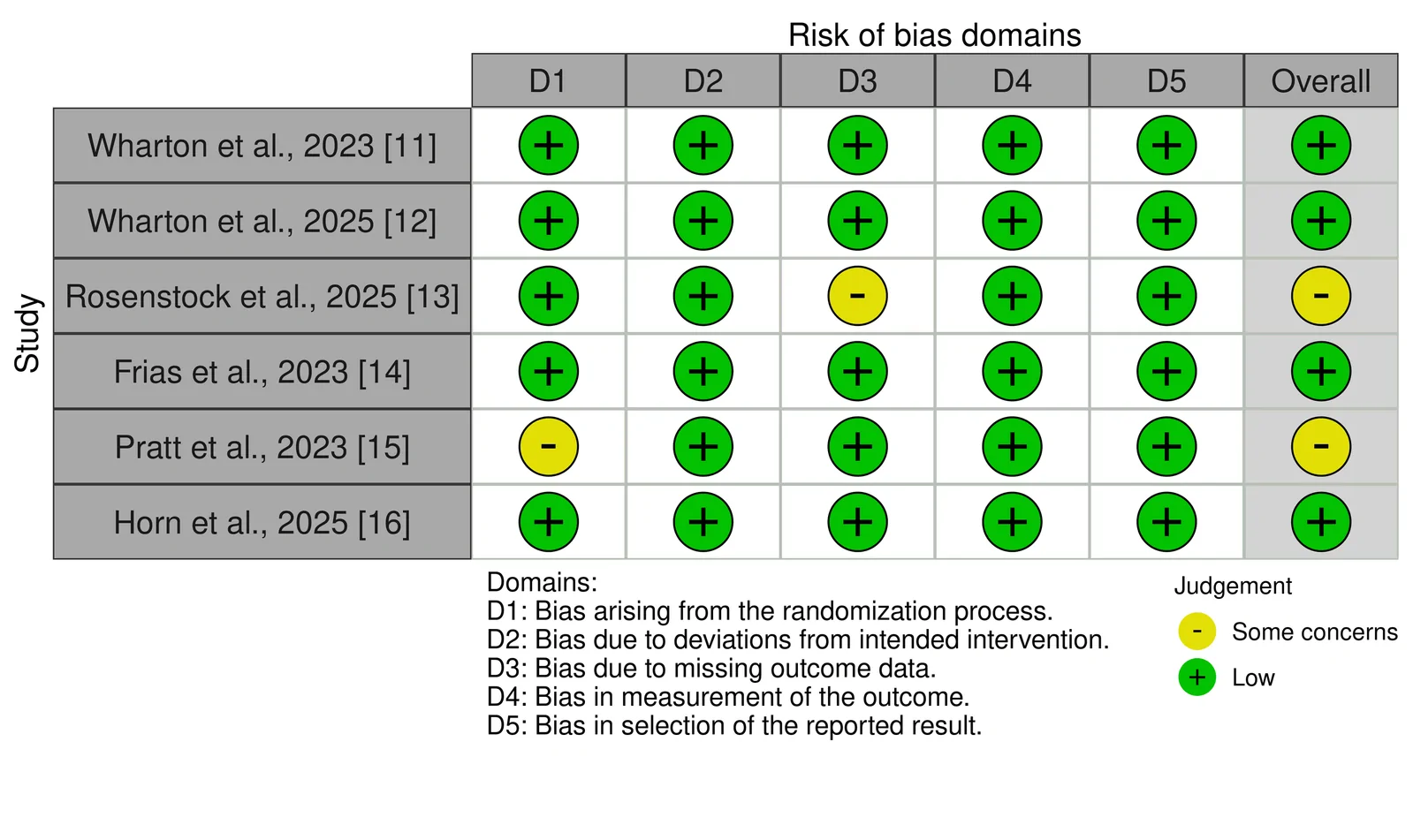
Risk of bias assessment. Risk of bias assessment of included randomized controlled trials using the Cochrane Risk of Bias 2 tool.

**Figure 3:**
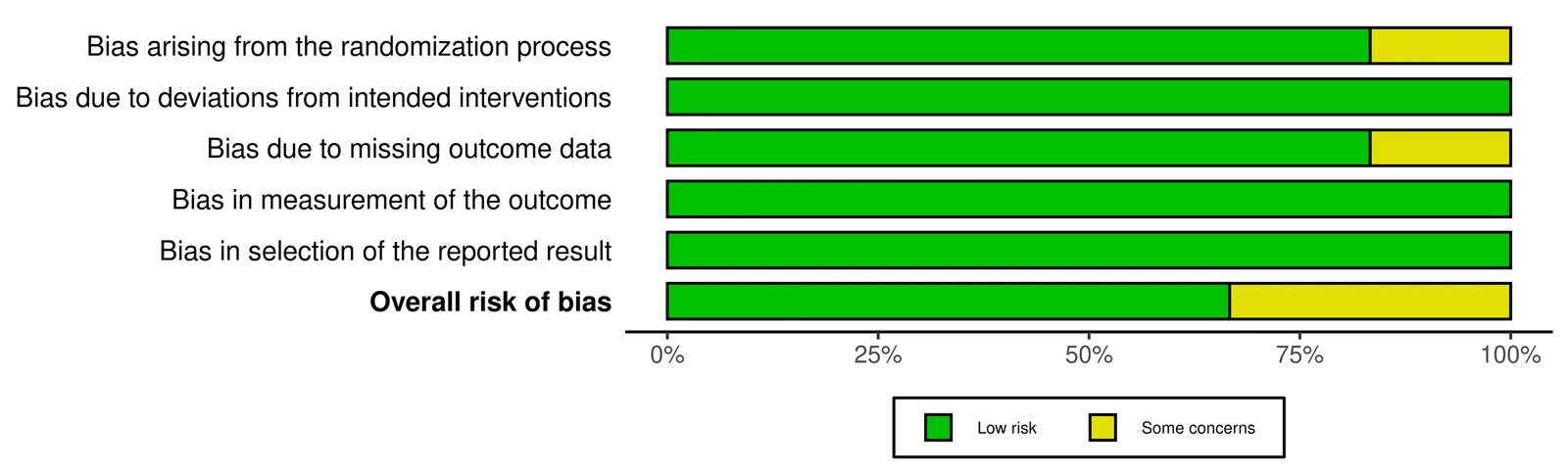
Summary plot of risk of bias across six included randomized controlled trials. Summary of risk of bias judgments across included randomized controlled trials using the Cochrane Risk of Bias 2 tool. The proportion of studies rated as low risk, some concerns, or high risk is presented for each bias domain.

The RoB 2 assessment evaluated five methodological domains: bias arising from the randomization process (D1), bias due to deviations from intended interventions (D2), bias due to missing outcome data (D3), bias in the measurement of outcomes (D4), and bias in the selection of the reported results (D5). Most studies demonstrated low risk across the evaluated domains, reflecting generally appropriate trial conduct and methodological consistency.

Wharton et al. (2023), Wharton et al. (2025), Frias et al. (2023), and Horn et al. (2025) were judged to have a low overall risk of bias [11,12,14,16]. In contrast, Rosenstock et al. (2025) and Pratt et al. (2023) were classified as having some concerns overall [13,15]. In Rosenstock et al., some concerns were identified regarding missing outcome data (D3), as body weight data were missing for a proportion of participants at week 40 and were addressed using prespecified imputation methods. In Pratt et al., some concerns were identified regarding the randomization process (D1), as baseline differences were observed across treatment groups, including differences in age, body weight, BMI, glycated hemoglobin (HbA1c), and duration of diabetes, in the context of the small sample size.

Overall, the methodological limitations identified were considered during interpretation of the findings.

Study Characteristics

Six randomized controlled trials involving a total of 6,022 participants evaluating oral orforglipron in adults with overweight or obesity, with or without type 2 diabetes, were included in the qualitative synthesis. The included studies were conducted by Wharton et al. (2023; n = 272) [11], Wharton et al. (2025; n = 3127) [12], Rosenstock et al. (2025; n = 559) [13], Frias et al. (2023; n = 383) [14], Pratt et al. (2023; n = 68) [15], and Horn et al. (2025; n = 1613) [16].

Study duration ranged from 12 to 72 weeks. Three studies included participants with type 2 diabetes, two included participants with obesity without diabetes, and one included participants with obesity and type 2 diabetes. All studies used randomized controlled trial designs and evaluated different oral orforglipron dose regimens compared with placebo, while one study additionally included dulaglutide as an active comparator.

Primary outcomes included body weight reduction, HbA1c improvement, and safety outcomes. Treatment protocols, dose-escalation strategies, and reported efficacy and safety endpoints varied across studies, contributing to clinical and methodological heterogeneity. Detailed study characteristics and main outcomes are summarized in Table 1.

**Table 1:** Data extraction. This table summarizes the main efficacy, cardiometabolic, glycemic, and safety outcomes reported across the included randomized controlled trials evaluating oral orforglipron. Abbreviations: AE, adverse event; CI, confidence interval; DXA, dual-energy X-ray absorptiometry; GI, gastrointestinal; HbA1c, glycated hemoglobin; HDL, high-density lipoprotein; hsCRP, high-sensitivity C-reactive protein; ITT, intention-to-treat; LDL, low-density lipoprotein; LSM, least-squares mean; NR, not reported; PD, pharmacodynamic; pp, percentage points; RCT, randomized controlled trial; SAE, serious adverse event; SBP, systolic blood pressure; SE, standard error; T2DM, type 2 diabetes mellitus; WL, weight loss.

Study	Population	N	Dose	Comparator	Follow-up	Analysis population/treatment estimand	Primary efficacy outcome	Placebo-adjusted effect (95% CI)	Weight-loss thresholds	Glycemic outcomes	Cardiometabolic outcomes	Safety outcomes	Statistical reporting
Wharton et al., 2023 [11]	Obesity without T2DM	272	12, 24, 36, or 45 mg once daily	Placebo	36 weeks; primary endpoint at week 26	Efficacy estimand; data collected before permanent treatment discontinuation	Body weight reduction of −8.6% to −12.6% at week 26, increasing to −14.7% at week 36	Week 26 placebo-corrected change: −6.5% to −10.6%; 95% CI for placebo-corrected effect: NR	≥10% WL: up to 75%; ≥15% WL: up to 48%	↓HbA1c, ↓fasting glucose	↓BMI, ↓waist circumference, ↓SBP, ↓triglycerides, ↓LDL, ↓non-HDL; reduction in fat mass by DXA	GI adverse events predominated; serious AEs reported in 7 participants; discontinuation due to AEs approximately 10–17%	95% CI reported; logistic regression and multiple imputation used
Wharton et al., 2025 [12]	Obesity without T2DM	3127	6, 12, or 36 mg once daily	Placebo	72 weeks	ITT population; treatment-regimen estimand	Body weight reduction of −7.5% to −11.2% at week 72	6 mg: −5.5 pp (95% CI −6.5 to −4.5); 12 mg: −6.3 pp (−7.3 to −5.4); 36 mg: −9.1 pp (−10.1 to −8.1)	≥10% WL: 33.3–54.6%; ≥15% WL: up to 36.0%	Prediabetes normalization; ↓fasting glucose	↓Waist circumference, ↓SBP, ↓triglycerides, ↓non-HDL cholesterol, ↓hsCRP, ↑HDL cholesterol	GI adverse events predominated; serious AEs: 3.8–5.5%; discontinuation due to GI AEs reported	P<0.001; 95% CI reported
Rosenstock et al., 2025 [13]	Early type 2 diabetes	559	3, 12, or 36 mg once daily	Placebo	40 weeks	All randomized participants; treatment-regimen estimand (ITT approach)	HbA1c reduction of −1.24% to −1.48%; body weight reduction of −4.5% to −7.6% at week 40	Body weight: 3 mg: −2.8 pp (95% CI −4.0 to −1.5); 12 mg: −4.1 pp (−5.4 to −2.7); 36 mg: −5.9 pp (−7.4 to −4.4)	≥10% WL: 15–30%; ≥15% WL: 4–10%	↓HbA1c, ↓fasting glucose; HbA1c <7% achieved in 68–73%	↓Triglycerides, ↓non-HDL cholesterol, ↓waist circumference, ↓SBP	GI adverse events predominated; serious AEs: 2.8–5.6%; discontinuation due to AEs: 2.2–5.7%	P<0.001 for prespecified comparisons; 95% CI reported
Frias et al., 2023 [14]	Type 2 diabetes	383	3, 12, 24, 36, or 45 mg once daily	Placebo; dulaglutide 1.5 mg once weekly	26 weeks	Efficacy analysis set; efficacy estimand using a hypothetical strategy	HbA1c reduction of −1.2% to −2.1%; body weight reduction of −3.7 to −10.1 kg	Body weight: 3 mg: −1.5 pp (95% CI −3.6 to 0.6); 12 mg: −4.4 pp (−6.5 to −2.4); 24 mg: −7.8 pp (−10.0 to −5.6); 36 mg: −7.4 pp (−9.4 to −5.4); 45 mg: −7.4 pp (−9.4 to −5.4) vs. placebo	≥10% WL: 8–48%; ≥15% WL: 4–24%	↓HbA1c, ↓fasting glucose	↓Waist circumference, ↓SBP; improved self-monitored glucose profiles, pulse rate increased	GI adverse events predominated; serious AEs: 1.8–10.6%; one mild pancreatitis event reported	95% CIs reported; P<0.0001 for primary placebo comparisons
Pratt et al., 2023 [15]	Type 2 diabetes	68	9, 15, 21, 27, or 45 mg once daily after dose escalation	Placebo	12 weeks	Participants receiving ≥1 dose; no formal treatment estimand reported	HbA1c reduction of −1.42% to −1.77%; body weight reduction of −0.24 to −5.77 kg	Body weight: approximately −4 to −6 kg vs. placebo for 9, 15, 27, and 45 mg; −0.75 kg for 21 mg; 95% CI NR	NR	↓HbA1c, ↓fasting glucose, ↓postprandial glucose	Delayed gastric emptying; appetite reduction; no significant lipid changes; pulse rate increased	GI adverse events predominated; no deaths or fatal SAEs; pancreatic enzyme elevations reported	Mixed-effects repeated-measures model; LSM ± SE reported for PD outcomes; descriptive statistics and 90% CIs reported
Horn et al., 2025 [16]	Overweight/obesity with type 2 diabetes	1613	6, 12, or 36 mg once daily	Placebo	72 weeks	Treatment-regimen estimand; all randomized participants regardless of intercurrent events	Body weight change: −5.1%, −7.0%, and −9.6% for 6, 12, and 36 mg, respectively; placebo −2.5%	6 mg: −2.7 pp (95% CI −3.7 to −1.6); 12 mg: −4.5 pp (−5.5 to −3.6); 36 mg: −7.1 pp (−8.2 to −6.1)	≥10% WL: 22.6–45.6%; ≥15% WL: up to 26.0%	↓HbA1c, ↓fasting glucose; HbA1c targets achieved more frequently	↓Waist circumference, ↓SBP, ↓non-HDL cholesterol, ↓triglycerides, ↓hsCRP, ↑HDL cholesterol	GI AEs predominated; discontinuation due to AEs: 6.1–10.6% vs. 4.1% placebo; pancreatitis and deaths reported	Model-based estimates; 95% CI; P<0.0001 for primary placebo comparisons

Results of individual studies

Weight Loss Outcomes

Across the included studies, treatment with orforglipron was consistently associated with clinically meaningful reductions in body weight, although the magnitude of effect varied according to study duration, dose, and diabetes status. A greater proportion of participants receiving orforglipron achieved clinically relevant weight-loss thresholds, including ≥5%, ≥10%, and ≥15% reductions, compared with placebo in studies reporting these outcomes. In Wharton et al. (2023) [11], conducted in adults with obesity without T2DM, mean body weight reduction at week 26 ranged from −8.6% to −12.6% compared with −2.0% with placebo, increasing further to −9.4% to −14.7% at week 36. Up to 75% of participants achieved ≥10% weight loss and up to 48% achieved ≥15% weight loss. Significant reductions in waist circumference were additionally observed, while dual-energy X-ray absorptiometry (DXA) analyses demonstrated that most weight reduction was attributable to fat mass loss. Similarly, in the phase 3 ATTAIN-1 trial by Wharton et al. (2025) [12], body weight reduction at week 72 ranged from −7.5% to −11.2% compared with −2.1% with placebo (p < 0.001), while 33.3% to 54.6% of participants achieved ≥10% weight loss and up to 36.0% achieved ≥15% weight loss. Among studies including participants with T2DM, Rosenstock et al. (2025) [13] reported body weight reductions ranging from −4.5% to −7.6% at week 40, with 15% to 30% of participants achieving ≥10% weight loss. Likewise, Frias et al. (2023) [14] demonstrated dose-dependent body weight reductions ranging from −3.7 kg to −10.1 kg, with ≥10% weight loss achieved in 8% to 48% of participants depending on dose. Pratt et al. (2023) [15], a shorter 12-week randomized phase 1b trial conducted in participants with T2DM, reported body weight reductions of up to −5.77 kg across orforglipron dose groups, although percentage-based weight-loss thresholds were not formally reported. Additionally, Horn et al. (2025) [16] demonstrated significant body weight reductions ranging from −5.1% to −9.6% at week 72 (p < 0.0001), with 22.6% to 45.6% of participants achieving ≥10% weight loss and up to 26.0% achieving ≥15% weight loss. Overall, greater reductions in body weight were generally observed in longer-duration studies, at higher treatment doses, and in populations without T2DM. These observations were descriptive and were not derived from formal between-group analyses.

Glycemic Outcomes (HbA1c Levels)

Glycemic outcomes were primarily evaluated in studies including participants with T2DM, with these studies demonstrating consistent improvements in HbA1c and fasting glucose levels. Rosenstock et al. (2025) [13] reported HbA1c reductions ranging from −1.24% to −1.48% compared with −0.41% in the placebo group (p < 0.001). Additionally, 68% to 73% of participants achieved HbA1c <7.0%, compared with 33% in the placebo group. Similarly, Frias et al. (2023) [14] demonstrated HbA1c reductions ranging from −1.2% to −2.1% compared with −0.4% with placebo and −1.1% with dulaglutide. HbA1c <7.0% was achieved in 65% to 96% of participants receiving orforglipron, with greater glycemic improvement observed at higher doses. In Pratt et al. (2023) [15], HbA1c reductions ranged from −1.42% to −1.77% across dose groups versus −0.40% with placebo. Despite the short follow-up duration, significant reductions in fasting plasma glucose and postprandial glucose exposure were observed across most treatment groups. Horn et al. (2025) [16] also demonstrated improvements in glycemic control, with HbA1c reductions ranging from −1.22% to −1.66% compared with −0.47% with placebo (p < 0.0001). HbA1c <7% was achieved in 64.6% to 75.5% of participants receiving orforglipron versus 30.5% with placebo, while HbA1c ≤6.5% was achieved in up to 66.6% of participants receiving higher-dose therapy. Overall, reductions in HbA1c, fasting serum glucose, and postprandial glucose levels were consistently observed across studies involving participants with T2DM.

Cardiometabolic Outcomes

Several studies additionally reported improvements in cardiometabolic parameters beyond weight reduction and glycemic control. Wharton et al. (2023) [11] demonstrated reductions in waist circumference, systolic blood pressure, triglycerides, low-density lipoprotein (LDL) cholesterol, total cholesterol, and non-high-density lipoprotein (non-HDL) cholesterol. Similar improvements in waist circumference, systolic blood pressure, triglycerides, non-HDL cholesterol, high-density lipoprotein (HDL) cholesterol, and inflammatory markers such as high-sensitivity C-reactive protein (hsCRP) were also reported in the phase 3 trials by Wharton et al. (2025) [12] and Horn et al. (2025) [16]. Rosenstock et al. (2025) [13] additionally reported reductions in triglycerides, waist circumference, fasting glucose, and systolic blood pressure after 40 weeks of treatment. Frias et al. (2023) [14] demonstrated improvements in waist circumference, systolic blood pressure, and self-monitored glucose profiles, although pulse rate increased modestly across treatment groups. Pratt et al. (2023) [15] did not identify significant lipid changes during the short study duration, although reductions in appetite scores, delayed gastric emptying, and significant increases in pulse rate were observed. These cardiometabolic parameters were reported as additional descriptive findings from the individual trials and were not subjected to formal cross-trial or treatment-duration comparisons.

Safety Outcomes

Adverse events were reported across all included studies, with gastrointestinal adverse events representing the most frequently reported safety outcome. In Wharton et al. (2023) [11], gastrointestinal symptoms, including nausea, constipation, vomiting, diarrhea, and eructation, were commonly reported and occurred more frequently at higher doses. Serious adverse events were reported among participants receiving orforglipron, while treatment discontinuation ranged from approximately 10% to 17%. Similarly, in the phase 3 ATTAIN-1 trial by Wharton et al. (2025) [12], gastrointestinal adverse events were generally mild-to-moderate and occurred primarily during dose escalation. Serious adverse events ranged from 3.8% to 5.5%, whereas discontinuation rates ranged from 5.3% to 10.3%. Among studies involving participants with T2DM, Rosenstock et al. (2025) [13] reported serious adverse events in approximately 2.8% to 5.6% of participants, while discontinuation rates ranged from 2.2% to 5.7%. No adjudication-confirmed pancreatitis or severe hypoglycemia events were identified. Frias et al. (2023) [14] reported treatment-emergent adverse events in 61.8% to 88.9% of participants receiving orforglipron, with serious adverse events ranging from 1.8% to 10.6% and discontinuation rates ranging from 11.8% to 19.1%. One adjudicated mild pancreatitis event was reported following treatment completion. In Pratt et al. (2023) [15], treatment-emergent adverse events occurred in 86.3% of participants receiving orforglipron compared with 41.2% receiving placebo. Gastrointestinal adverse events predominated, while no deaths or fatal serious adverse events were reported. Eleven discontinuations occurred overall, including seven among participants receiving orforglipron. Pulse rate increased across treatment groups, and two participants developed clinically significant elevations in pancreatic enzymes. Finally, Horn et al. (2025) [16] did not identify a major unexpected safety signal, although gastrointestinal symptoms remained the most common adverse events and occurred more frequently during dose escalation. Serious adverse events occurred in 9.2% of participants overall, with discontinuation rates ranging from 6.1% to 10.6%. Ten deaths were reported during the study period, although most were considered unrelated to study treatment. Adjudication-confirmed pancreatitis occurred in one participant receiving orforglipron and two placebo-treated participants. Overall, adverse events were frequent but generally manageable across studies, with gastrointestinal symptoms and treatment discontinuation occurring more commonly in higher-dose groups.

Overall Findings

Collectively, the included studies demonstrated consistent reductions in body weight among participants receiving orforglipron across diabetic and non-diabetic populations. Greater weight reduction was generally observed in longer-duration studies and at higher treatment doses. Improvements in glycemic control were also consistently reported among studies including participants with T2DM, particularly regarding reductions in HbA1c, fasting glucose, and achievement of glycemic targets.

Cardiometabolic improvements, including reductions in waist circumference, systolic blood pressure, triglycerides, non-HDL cholesterol, inflammatory markers, and improvements in HDL cholesterol, were additionally reported in several phase 2 and phase 3 trials.

Safety findings were generally consistent across studies, with gastrointestinal adverse events representing the most commonly reported adverse events. Although higher-dose groups frequently demonstrated greater rates of gastrointestinal symptoms and treatment discontinuation, no major unexpected safety signals were consistently identified across studies.

Discussion

Oral GLP-1 receptor agonists such as orforglipron represent a promising therapeutic approach for obesity and type 2 diabetes, with oral administration representing a potential practical alternative to injectable therapies. Previous studies have suggested that many patients prefer oral alternatives for chronic disease management regardless of the underlying condition. Current evidence from the included studies suggests that orforglipron may be associated with clinically meaningful reductions in body weight in adults with overweight or obesity [11-16].

Across studies reporting body weight change as a percentage, reductions ranged from approximately −4.5% to −14.7%, while the proportion of participants achieving ≥10% weight loss ranged from 8% to 75% depending on study duration, dose, and diabetes status. Greater weight reduction was generally observed in longer-duration studies and among participants without T2DM. Similar findings have been reported with other GLP-1 receptor agonists, including tirzepatide, in which non-diabetic populations appeared to achieve greater weight reduction compared with diabetic populations [17].

Dose-response patterns across studies suggest that higher doses of orforglipron were associated with greater reductions in body weight and HbA1c levels. Gastrointestinal adverse events and treatment discontinuation became more frequent at higher doses. However, given the indirect comparisons across heterogeneous trials, the available evidence does not allow conclusions regarding an efficacy plateau or the optimal dose of orforglipron.

Improvements in glycemic control were also consistently observed across studies involving participants with T2DM. HbA1c reductions generally ranged from approximately −1.2% to −2.1%, with several studies additionally reporting reductions in fasting glucose, postprandial glucose, waist circumference, systolic blood pressure, triglycerides, and non-HDL cholesterol. These findings suggest that orforglipron may provide additional cardiometabolic benefits beyond weight reduction alone.

Gastrointestinal adverse events, including nausea, vomiting, diarrhea, and constipation, represented the most commonly reported adverse events across studies and were generally more frequent during dose escalation. Higher-dose groups also demonstrated greater rates of treatment discontinuation in several trials. Nevertheless, most gastrointestinal events were mild-to-moderate in severity, and no major unexpected safety signals were consistently identified. These findings are generally consistent with the established safety profile of GLP-1 receptor agonists reported in previous obesity and diabetes studies [18].

The findings of the present review are broadly consistent with previous systematic reviews and meta-analyses evaluating orforglipron. A recent meta-analysis of five randomized controlled trials similarly reported reductions in body weight and improvements in glycemic and lipid parameters, with dose-dependent effects observed across several efficacy outcomes; gastrointestinal adverse events were more frequent with orforglipron than with placebo [19]. An earlier meta-analysis of four randomized controlled trials also reported reductions in body weight, BMI, and waist circumference with once-daily oral orforglipron, together with a greater proportion of participants achieving clinically relevant weight-loss thresholds compared with placebo [20]. The certainty of evidence in that review was rated as low for body weight and very low for several other outcomes, primarily because of risk of bias, inconsistency, and imprecision [20]. Overall, these findings are consistent with the general efficacy and safety patterns observed in the present review, although differences in study populations, treatment duration, and available evidence should be considered when comparing findings across reviews.

Beyond weight and metabolic outcomes, a recent systematic review and meta-analysis evaluating GLP-1 receptor agonists reported a reduction in Alcohol Use Disorders Identification Test (AUDIT) scores and described reductions in other alcohol-related measures, including drinking and craving outcomes [21]. However, the review included both randomized and observational evidence, with substantial heterogeneity across studies. Importantly, these findings were derived from GLP-1 receptor agonists other than orforglipron and therefore should not be extrapolated directly to orforglipron. They provide complementary evidence regarding potential effects of GLP-1 receptor agonism beyond weight reduction and glycemic control [21].

Several limitations should be considered when interpreting these findings. The included studies demonstrated substantial heterogeneity regarding study duration, dose-escalation strategies, comparator groups, and reported outcomes, which precluded quantitative meta-analysis. In addition, one included study was a shorter phase 1b trial with a relatively small sample size, and all included trials were funded by Eli Lilly and Company. Follow-up duration also varied considerably across studies, limiting assessment of long-term efficacy and safety. Comparisons across trials were indirect and descriptive and should therefore be interpreted cautiously. Direct head-to-head evidence comparing orforglipron with other obesity pharmacotherapies remains limited, restricting conclusions regarding comparative efficacy and safety. Furthermore, the available studies provide limited evidence regarding long-term cardiovascular outcomes.

Overall, current evidence suggests that oral orforglipron may represent an effective therapeutic option for weight reduction and glycemic improvement in adults with overweight or obesity, including populations with T2DM. However, additional long-term randomized controlled trials are needed to further clarify optimal dosing strategies, long-term safety, and sustained clinical effectiveness.

## Conclusions

Current evidence suggests that oral orforglipron may be associated with clinically meaningful weight reduction and improvements in glycemic control among adults with overweight or obesity, including populations with T2DM. Overall, the available studies suggest that orforglipron may represent a promising oral therapeutic option for obesity and weight management; however, substantial heterogeneity across study populations, treatment duration, and reported outcomes limits direct comparison between trials. Additional long-term randomized controlled trials and head-to-head comparative studies are needed to better define the optimal dosing strategy, long-term safety profile, and clinical role of orforglipron in obesity management.
